# **Hypnosis Antenatal Training for Childbirth (HATCh): a randomised controlled trial **[NCT00282204]

**DOI:** 10.1186/1471-2393-6-5

**Published:** 2006-03-05

**Authors:** Allan M Cyna, Marion I Andrew, Jeffrey S Robinson, Caroline A Crowther, Peter Baghurst, Deborah Turnbull, Graham Wicks, Celia Whittle

**Affiliations:** 1 Dept. Anaesthesia, Women's and Children's Hospital, Adelaide SA 5006, Australia; 2 Dept. Obstetrics and GynaecologyUniversity of AdelaideWomen's and Children's HospitalSA 5006, Australia; 3 Dept. Public Health,3^*rd *^floor Norwich buildingWomen's and Children's Hospital, Adelaide SA 5006, Australia; 4 Dept. Psychology University of AdelaideFrome RdSA 5006, Australia; 5 ^5^Dept. Psychological MedicineWomen's and Children's Hospital, Adelaide SA 5006, Australia; 6 Fountain Corner Family Practice,57 Unley Rd, Parkside, Adelaide 5063, Australia

## Abstract

**Background:**

Although medical interventions play an important role in preserving lives and maternal comfort they have become increasingly routine in normal childbirth. This may increase the risk of associated complications and a less satisfactory birth experience. Antenatal hypnosis is associated with a reduced need for pharmacological interventions during childbirth. This trial seeks to determine the efficacy or otherwise of antenatal group hypnosis preparation for childbirth in late pregnancy.

**Methods/design:**

A single centre, randomised controlled trial using a 3 arm parallel group design in the largest tertiary maternity unit in South Australia. Group 1 participants receive antenatal hypnosis training in preparation for childbirth administered by a qualified hypnotherapist with the use of an audio compact disc on hypnosis for re-enforcement; Group 2 consists of antenatal hypnosis training in preparation for childbirth using an audio compact disc on hypnosis administered by a nurse with no training in hypnotherapy; Group 3 participants continue with their usual preparation for childbirth with no additional intervention. Women > 34 and < 39 weeks gestation, planning a vaginal birth, not in active labour, with a singleton, viable fetus of vertex presentation, are eligible to participate. Allocation concealment is achieved using telephone randomisation. Participants assigned to hypnosis groups commence hypnosis training as near as possible to 37 weeks gestation. Treatment allocations are concealed from treating obstetricians, anaesthetists, midwives and those personnel collecting and analysing data. Our sample size of 135 women/group gives the study 80% power to detect a clinically relevant fall of 20% in the number of women requiring pharmacological analgesia – the primary endpoint. We estimate that approximately 5–10% of women will deliver prior to receiving their allocated intervention. We plan to recruit 150 women/group and perform sequential interim analyses when 150 and 300 participants have been recruited. All participant data will be analysed, by a researcher blinded to treatment allocation, according to the "Intention to treat" principle with comprehensive pre-planned cost- benefit and subgroup analyses.

**Discussion:**

If effective, hypnosis would be a simple, inexpensive way to improve the childbirth experience, reduce complications associated with pharmacological interventions, yield cost savings in maternity care, and this trial will provide evidence to guide clinical practice.

## Background

Pain during labour and childbirth represents a complex interaction of multiple physiological and psychological factors [1]. Techniques such as epidural analgesia, have been shown to be the most effective form of pain relief in labour[2] but, can deprive the mother of an optimal birth experience[3] and are associated with adverse effects such as post-dural puncture headache and neurological injury [4,5]. Although, long term sequelae are rare, such complications can be debilitating and extremely distressing when they occur [6]. All pharmacological interventions cross the placenta to some degree which leads to concerns of adverse effects on the fetus. The recent Australia and New Zealand College of Anaesthetists (ANZCA) working party report emphasises that non-pharmacological treatment options should be considered before analgesic medications are used particularly just before delivery [1]. Women's desires for and expectations of pain relief during labour and delivery vary widely [7] and high quality pain relief does not necessarily equate with a high level of satisfaction [8]. The increasing medicalisation of childbirth [9] has led many women to look for alternative means of relieving labour pain [10].

Fear, anxiety and maternal feelings of a loss of control frequently play a role in the incidence and intensity of pain during childbirth and are associated with an increased risk of post-traumatic stress disorder[11] and postnatal depression [12,13]. Hypnosis is a psychological intervention that has been shown to provide analgesia and reduce anxiety and in the peri-operative setting [14-16] and since the mid 1980s has been advocated as a useful tool in the management of depression [17,18]. Claims that hypnosis is a safe and valuable tool in pregnancy and childbirth [19-21] is supported by numerous reports in the literature describing the successful use of hypnosis as an analgesia adjunct during childbirth. [22-24] For many years hypnosis has suffered greatly from misunderstanding and prejudice [25]. However, more recently the use of clinical hypnosis has become an area of increasing clinical interest and research [26-28] Advances in neuro-imaging have led to an understanding of the neuro-physiological changes occurring during hypnosis induced analgesia [29]. The anterior cingulate gyrus has been repeatedly demonstrated, by positron emission tomography, to be one of the sites in the brain affected by hypnotic modulation of pain. [29-31] The suppression of neural activity, between the sensory cortex and the amygdala – limbic system, appears to inhibit the emotional interpretation of sensations being experienced as pain. Hypnosis appears to be a state of narrow focused attention, reduced awareness of external stimuli, and an increased response to suggestions [32,33]. Suggestions are verbal or non-verbal communications that result in apparent spontaneous changes in perception or behaviour. These therapeutic communications are directed to the patient's subconscious and the responses are independent of any conscious effort or reasoning [34]. Potentially, medical hypnosis could be used alone for pain relief as part of a woman's care during childbirth. In practice however, hypnosis is best seen as an adjunct to facilitate and enhance other analgesics. The well recognised problems associated with current analgesia techniques and the increasing medicalisation of childbirth has led many women to look for an alternative means of relieving pain in labour [10]. Bonica estimates that up to 25% of women obtain complete analgesia when using hypnosis for pain relief in labour [35]. The responsiveness of women to hypnosis appears to be increased in pregnancy and in primiparous when compared with multiparous women [36,37]. A wide variety of personnel have used hypnosis effectively including medical students, [38] psychologists, [39,40] hypnotherapists[37] and obstetricians [41,42].

Systematic review evidence suggests that learning hypnosis techniques for use in childbirth would allow mothers to reduce their need for pharmacological analgesia, and other interventions such as intravenous oxytocics, and increase their chance of having a spontaneous vaginal birth[10,22,43]. Until recently, evidence of the effectiveness of hypnosis as an analgesia adjunct during childbirth was limited to three small trials being of adequate quality for meta-analysis. In addition the maternal populations under investigation did not have access to an "on demand" epidural service for labour analgesia which is widely available in many developed countries. A recent large study from the USA investigating the preparation of women for childbirth using hypnosis in the 1st trimester[44] will soon be available for inclusion in an updated meta-analysis. Neither the intervention nor the number of sessions were standardised in this study which was performed over a ten year period in the USA. Such features of previous studies limit the reproducibility of the intervention and decrease external validity.

Since April 2002, we have been developing an antenatal hypnosis training program for women in late pregnancy (after 35 weeks gestation) to be utilised for anxiolysis and as an analgesia adjunct during childbirth. Initially, we were seeing women on an individual basis. However increasing demand for hypnosis preparation for childbirth from mothers, midwives and obstetricians at our institution has led us to our current practice of training groups of 5–10 women/week in self hypnosis techniques developed along the lines described by Waxman, [45]. McCarthy [21,46] and Bjenke [24]. The hypnosis training program has continued to develop over the last three years utilising advice from senior clinical hypnotherapists in Australia and New Zealand with expertise and substantial experience of preparing over 1000 women in hypnosis preparation for childbirth. The intervention lasts approximately one hour and the hypnosis sessions are held for three consecutive weeks. Birth outcomes of 77 antenatal women taught hypnosis in preparation for childbirth between January 2003 and August 2004 were compared with parity matched controls delivering after 37 weeks gestation during 2003 at our institution. Primiparous women, receiving hypnosis preparation, used fewer epidurals than controls 18/50 (36%) vs 765/1436 (53%) (RR0.68, 95%CI 0.47,0.98) and less augmentation 9/50 (18%) vs 523/1436 (36%) (RR 0.48, 95%CI 0.27,0.90) [47]. These findings are consistent with those of our systematic review [22].

### Number of hypnosis sessions

Although most clinical hypnotherapists use three or more sessions antenatally when training women with hypnosis preparation for childbirth, Rock et al found hypnosis effective in untrained mothers during their labour [38]. Our clinical experience at our own institution suggests that the intervention is optimally delivered when three sessions are scheduled in late pregnancy. Interestingly, despite differences between trials in the timing and number of hypnosis interventions reported, outcomes are consistently in favour of hypnosis [22]. However, this could simply reflect possible publication bias.

### Timing of the intervention

Tiba suggests that as pregnancy progresses responsiveness to hypnosis and suggestion increases [36]. Our experience over the last three years has found that the vast majority of women have little difficulty learning this technique in the last four weeks of pregnancy.

### Groups vs individual administration of hypnosis

Leeb successfully used hypnosis in groups of up to 20 women in preparation for childbirth[48] while Harmon demonstrated a range of beneficial outcomes following antenatal hypnosis training in groups of 15 women [39]. Our own experience suggests that group hypnosis is effective and allows far more women to receive the intervention than would be the case with individual administrations. Some practitioners claim that an individualised approach is more effective but this has not been shown in a study of the effectiveness of hypnosis in treating hyperemesis [49].

#### Multiparous vs nulliparous

Previous randomised comparisons of hypnosis in this setting have investigated nulliparous women only. Two hypnosis studies investigating multiparous women used parity matched controls. These reports show similar (but reduced) treatment effects in favour of hypnosis [37,47].

#### Evidence of the effectiveness of hypnosis in the management and prevention of anxiety and postnatal depression

Hypnosis has recently becoming advocated as a useful non-pharmacological intervention in the treatment of depression [17,50]. There are several reports of a low incidence of postnatal depression associated with women preparing for childbirth using hypnosis techniques although comparative data is lacking [23,46] In addition, there is convincing evidence in the peri-operative setting that the use of hypnosis decreases patient anxiety and reduces overall costs [28,51,52].

#### Safety of hypnosis in childbirth

There are two published reports of a complication of hypnosis associated with an obstetric patient. One involved a parturient prior to labour exhibiting psychotic symptoms believing that she had been assaulted, [53] and the other involved a treatable post partum anxiety and compulsive behaviour associated with the use of hypnosis during labour [54]. Other reported problems with the use of (non obstetric) medical hypnosis in the literature have been mainly associated with the use of age regression techniques, by inexperienced practitioners, or in patients with psychoses [53]. It has been recommended that hypnosis should be used by practitioners within their field of expertise [55]. This is consistent with the view of a British Medical Association report confirming the relevance and appropriateness of the use of hypnosis by obstetricians and anaesthetists [56]. The mythology surrounding hypnosis that it is too time consuming, limits free will or induces amnesia of the birth experience are dispelled both by Werner[53] and more recently Nash [57]. There appears to be little basis for the fears surrounding these supposed dangers of hypnosis in obstetrics, although such opinions may have been a deterrent to its application [53].

### Supplementing hypnosis using an audio compact disc (cd) at home and in labour

Several workers ask patients to listen to an audio tape of hypnosis suggestions at home as practice in their preparation for childbirth re-enforcing the techniques learned in the classroom [39,46]. The heterogeneity seen in our systematic review[22] can be explained by the use of supplemental tapes of suggestions, in one of the studies, in addition to live preparation [39] This appears to support the view that it is beneficial for subjects to practice the intervention using taped suggestions at home [21]. There are however no randomised studies to confirm whether listening to hypnotic suggestions, on an audio tape or audio compact disc (cd), is of additional value. However the use of a tape or audio cd for re-enforcing the suggestions is a simple, cheap supplement to our hypnosis sessions that allows the intervention to be standardised and maximises external validity. The effectiveness of standardised over individualised suggestions during hypnosis has been studied previously [58]. If the audio cd is shown to be an effective intervention it could be easily implemented in other hospitals with no experience of hypnosis preparation for childbirth.

### Development of the structured intervention delivered by audio cd on hypnosis

Audio cds on hypnosis were developed in our institution following increasing requests from patients for a supplement to what was learned in the live hypnosis sessions. An experienced physician who has practiced full time hypnotherapy for over 10 years and whose practice involves regular hypnotherapy preparation for childbirth observed our hypnosis group sessions for several weeks and took notes of the types of suggestions utilised during each session. Our antenatal hypnosis training sessions are based on published scripts of suggestions, by experts in the administration of hypnosis in childbirth, combined with our own clinical experience. A final written script for each session based on our current clinical practice in training women using hypnosis in preparation for childbirth was agreed by the hypnotherapist members of our research team. These scripts were used to produce three audio cds that mirrored our current hypnosis preparation for childbirth training program. The audio cds were produced at a local recording studio and each lasted between 21 and 32 minutes. A 4th audio cd lasting 18 minutes has also been developed for use during labour and childbirth. Multiple copies were made through our institution's digital media department. The cds are labelled with a caution that they should not be used while operating machinery or driving. Two lead investigators' names (AMC and MIA) and contact phone numbers of our institution are shown on each cd label. Participants were advised that the cds are for their use alone as part of the HATCh trial.

Although there is evidence of the effectiveness of hypnosis for labour analgesia, too few women have been rigorously investigated in late pregnancy and trial heterogeneity prevents clear recommendations of what constitutes an effective intervention. Further adequately powered well-designed trials are required.

This trial seeks to determine the efficacy or otherwise of antenatal group hypnosis preparation for childbirth in late pregnancy.

### The specific aims of this study are to assess whether antenatal hypnosis preparation for childbirth:

1. is an effective way of reducing maternal use of pharmacological analgesia

2. reduces the incidence of adverse effects on the mother

3. reduces the incidence of adverse affects on the baby

4. impacts on the mother's emotional well being

5. is cost effective

### Additional specific aims

6. To compare two methods of delivering antenatal group hypnosis in a double blind fashion, one method using a hypnotherapist to deliver the intervention followed by an audio cd on hypnosis for re-enforcement of the techniques learned. The other method is to use the an audio cd on hypnosis alone administered by a nurse with no hypnotherapy training.

**[see **Additional file 1**] **for detailed hypotheses

## Methods/design

### Design of the study

A single centre, randomised controlled trial using a 3 arm parallel group design to assess the effects of hypnosis preparation for childbirth in late pregnancy as a means of reducing analgesia requirements in labour and improving other birth outcomes.

Group 1 participants receive antenatal hypnosis training in preparation for childbirth administered by a qualified hypnotherapist with the use of audio compact discs on hypnosis for re-enforcement; Group 2 consists of antenatal hypnosis training in preparation for childbirth using audio compact discs on hypnosis administered by a nurse with no training in hypnotherapy; Group 3 participants continue with their usual preparation for childbirth with no additional intervention.

The study setting is the largest tertiary maternity unit in South Australia. Participant inclusion criteria are women > 34 and < 39 weeks gestation, with a singleton, viable fetus, vertex presentation, who are not in active labour (active labour is defined as cervical effacement and dilatation associated with regular uterine contractions) and who are planning a vaginal birth. Exclusion Criteria are: previous hypnosis preparation for childbirth; poor understanding of English requiring a translator; women who are already enrolled in another pregnancy trial where analgesia requirements are an outcome measure; active psychological or psychiatric problems such as: active depression requiring treatment by a psychiatrist; schizophrenia; prior psychosis; severe intellectual disability. Women with pain caused by specific pathological entities such as: congenital neuromuscular disorders; spina bifida; metastatic disease; osteoporosis; rheumatoid arthritis; fractures, were also excluded.

### Interventions and comparisons

Potentially eligible women > 34 weeks gestation will be identified during their attendance at the antenatal clinic, antenatal classes, or midwifery group practice, or while an in-patient on the antenatal ward. Posters advertising the trial will be placed around the hospital in these areas. An "expression of interest" form will be made available where mothers can obtain contact information from our research coordinator and advised further about what is involved if participating in the trial. All women approached for eligibility will have a structured explanation regarding participating in the trial and numbered as per consort statement [59]. Potential trial participants are informed that they have a 66% chance of being allocated to one of the hypnosis interventions and a 33% chance of proceeding with their childbirth preparation as per the usual practice of our institution. Eligible women who decline to enter the trial will be asked for consent to allow us to collect routine birth outcome data without any other intervention. Reasons for declining to participate will be recorded as: not interested, against my religion, too busy, don't believe in it, declined randomisation, other. Other reasons for leaving the study prior to randomisation will be noted. For safety considerations the consent process will include permission from participants to inform their GP and obstetrician, if further clinical assessment or treatment for postnatal depression is indicated. All eligible women approached who decide to continue their preparation for childbirth outside the trial will be asked permission for us to collect birth outcome data so that we can compare primary outcomes of non participants with those of women randomised to our "no intervention" control group.

Study participants will have baseline demographic data collected including parity, health insurance status, highest level of formal education, marital status and the use of any complementary therapies such as acupuncture or yoga. At this time participants will be asked to complete Spielberger Stait/Trait anxiety[60] and Edinburgh post-natal depression scales[61]. In addition they will be tested for hypnotisability using the Creative imagination Scale (CIS). We deliver the scale by asking participants to listen to a standardised audio CD of the CIS in the presence of a researcher. Participants then complete an answer sheet detailing their imaginative experience on a 5 point Likert scale [62].

### Randomisation

Biomedical statisticians at our institution's Dept. of Public Health have arranged for a computer generated random number sequence, of unspecified block size stratified for parity. This randomisation plan will produce study arms of approximately equal size with similar numbers of nulliparous and multiparous participants.

### Allocation concealment

We will be provided with the group allocation for each individual participant via telephone or a networked computer program. Allocation concealment is assured as the participant ID, parity and eligibility for trial entry is confirmed and recorded centrally prior to verifying a participant's group allocation. Participants assigned to hypnosis groups are to be given appointments by our research assistant to commence antenatal group hypnosis training as near as possible to 37 weeks gestation.

### Consort criteria

**Figure 1:**
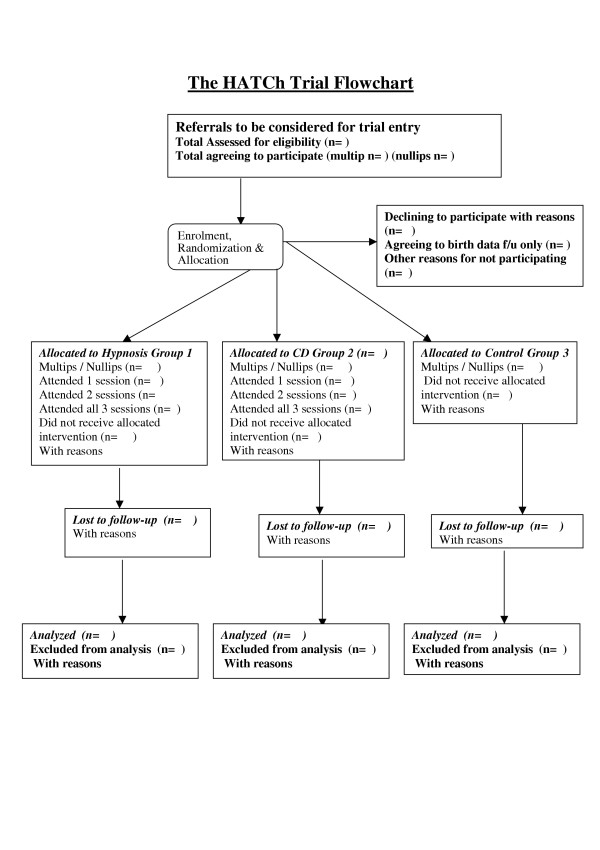
**Title. **HATCh Trial Flow

All eligible women will be given a trial identification number as per consort statement (See Figure 1 Trial Flow)

### Ethics approval

Local Regional Ethics Committee approval has been obtained prior to recruitment. Recruitment commenced in December 2005.

### Trial registration

This trial has been registered with the Australian Clinical Trials register (ACTRN012605000018617) December-05 and at clinicaltrials.gov (NCT00282204) January-06.

### Treatment schedules

A researcher is responsible for base line data management, collection and co-ordinating appointments with the hypno-therapist and nurse supervising Group 1 and 2 participants respectively, directing women to their allocated interventions. Following baseline demographic data collection which includes the administration of the Edinburgh Postnatal Depression Scale (EPDS)[61], Spielberger[60] and Creative Imagination Scale (CIS)[62], trial participants are informed of where and when to attend their allocated sessions. The two interventions are administered in groups of up to ten women at our institution's group physiotherapy room. Participants allocated to an intervention are requested to attend sessions which are scheduled as near as possible to 37 weeks gestation and are held at weekly intervals for three consecutive weeks. Those women having a planned induction of labour will be allocated their hypnosis sessions as near as possible to commence within three weeks of the induction date rather than the original expected date of confinement (EDC).

### Group 1: Antenatal hypnosis plus hypnosis audio CD (hypnotherapist guided)

#### Session 1

Discussion about fears and anxieties. Questions about hypnosis answered, Seeding expectancy of success. Basic hypnosis taught using progressive relaxation with a self hypnosis component at the end. Session 1 cd played and women asked to practice this on a daily basis until the next session.

#### Session 2

After a hypnosis induction, suggestions are given for confidence, coping and strength during contractions. Women are asked to focus on breathing for analgesia and relaxation. Suggestions for time distortion: this allows contractions to seem shorter and rest periods between contractions longer than they really are. Standardised suggestions given for a labour rehearsal involving recurrent fractionation and staircase imagery[21]. Session 2 section of cd played and women asked to practice this on a daily basis until the next session.

#### Session 3

Pain control techniques – Amnesia suggestion for unhelpful comments and utilisation of helpful suggestions. Dissociation and lower body anaesthesia elicited using the lignocaine spa imagery21 Suggestions for uterine contraction after delivery and relaxation to facilitate breastfeeding. Session 3 audio cd played and women asked to practice hypnosis while listening to the whole cd on a daily basis until the birth. Women told that they can listen to the labour audio cd during labour if they wish.

### Group 2: Antenatal hypnosis audio CD (nurse guided)

#### Session 1

Structured information given regarding getting the most from the cds. An explanation is given regarding the structure of the sessions and how to obtain most benefit when listening to the cd. It is suggested not to try to pay conscious attention and to go along with any instructions given will provide optimum benefit. Questions answered and Session 1 audio cd played. Participants are asked to listen to the cd on at least a daily basis until the next session.

#### Session 2

Questions answered. Session 2 audio cd played. Participants are asked to listen to the cd on at least a daily basis until the next session.

#### Session 3

Questions answered. Session 3 audio cd played. Participants are asked to listen to the cd on at least a daily basis until the next session.

### Group 3: No intervention control

Women allocated to usual care will not be asked to attend any further sessions at the hospital other than those required for their usual antenatal care. The next involvement of these mothers will be in responding to a maternal questionnaire in the early postnatal period.

### Compliance with treatment schedules

Those women allocated to attend either of the two intervention groups are asked to listen to each session audio cd on a daily basis between the weekly sessions. Those participants who are unable to attend one or more sessions in person will be contacted by telephone to confirm all is well. We will ask the participant if we could post them the audio cd(s) of the missed session(s) for them to listen to prior to their next hospital visit. Trial participants will be able to withdraw at any stage of the trial. All randomised participants and their babies will receive follow-up in the post partum period in an identical manner regardless of the treatment actually received.

### Care during labour and the postnatal stay

This will be managed by the trial participant's attending midwife, obstetrician and neonatal team as per the usual practice of our institution.

#### Power calculation

It is proposed that sufficient women be randomised to provide reliable evidence of the effects of antenatal hypnosis regarding the primary outcome measure of this study: the incidence of not using pharmacological pain relief during labour. An audit of 100 consecutive mothers birthing at the Women's and Children's Hospital in May 2004 showed an incidence of using one or more pharmacological interventions of 80%. In order to show a clinically relevant fall of 20% in the number of women requiring pharmacological analgesia ie from 80% to 64%. Using a 2 tailed calculation (Nquery advisor computer program V.5) a study with 80% power would require each group to contain 135 women to detect this difference at the 0.05 level.

Complete collection of the primary endpoint is likely as all analgesia techniques used such as Entonox, pethidine and epidural analgesia are documented routinely in the labour ward birth register and medical record by the woman's treating midwife. We plan to recruit 150 women/group as we estimate that 10% of participants' will deliver prior to receiving their allocated treatment. The findings of our case matched control study at the Women's and Children's Hospital[47] are consistent with those of our systematic review of the literature on this topic [22]. No comparative trials have investigated the effects of hypnosis on the incidence of postnatal depression although acknowledged experts in the field report a low incidence in women taught hypnosis (<1%) [21]. The prevalence of post-natal depression in several recent studies in Australia is between 13 and 18% [63]. Our proposed study has a power of 80% at the 0.05 level of significance to show a reduction from 16% to 5.4% for this secondary outcome. For normally distributed continuous measures (eg EPDS) we would be able to detect shifts of 0.4 SD with a power of 80 % between individual arms of the study (n = 135 per group). Comparisons which exploit the two treatment groups (assuming a conservative estimate of postnatal data in 270 subjects in Groups 1 and 2) would have 80% power to detect a shift of only 0.28 SD.

**[see **Additional file 2**] **for estimated detectable differences in key endpoints with sample size of 135/group. Based on these estimates a total recruitment of 450 women will enable detection of significant differences of clinical relevance for the primary outcome and for some key secondary outcomes. We recognize that a trial of this size may be too small to detect differences in the risk of some of our secondary endpoints but the information will allow comparisons of women in other hypnosis studies in a systematic review or suggest other beneficial outcomes or adverse effects that require further controlled evaluation.

### Data collection and outcomes

Our primary outcome the use of pharmacological analgesia during labour and childbirth will be collected from the birth register where all analgesia is documented by the attending midwife. Any unclear entries will be clarified by referring to the medical and midwifery record of the birth. Most of our key secondary endpoints such as the use of oxytocics, the mode of delivery, neonatal Apgar score at 5 minutes < 7 and maternal admission to the High Dependency Unit (HDU) or the Intensive Care Unit (ICU) will be obtained from the birth register. These outcomes will have been documented by the attending midwife who will be unaware of the parturient's group allocation. A second researcher who is blinded to treatment allocation will manage data collections on the ward and from post-natal questionnaires. Details of maternal side effects will be collected such as; PPH=>600 mls; blood transfusion; death; ICU admission, meconium stained liquor; babies admitted to neonatal unit. Mothers will be asked to complete a postpartum questionnaire while in hospital and asked to rate the overall pain experienced during labour and childbirth, whether the birth experience was: Worse/better/same as expected, whether they felt in control during the labour during the birth and whether the birth was rated a positive or negative experience. We will also ask: how well the mother felt she coped with labour and childbirth; whether hypnosis training was obtained outside the trial; and whether hypnosis will be used in future pregnancies. Length of neonatal nursery stay, length of maternal stay in hospital and the number of women breast feeding at discharge from hospital will also be recorded.

#### Further follow up

Postnatal questionnaires will be sent to each participant at 6 weeks and 6 months after the birth where the EPDS[61] and Spielberger anxiety scales[60] will be repeated. Women will be asked whether they are still breast feeding and invited to make comments of any problems or difficulties with the intervention. Two weeks after postage of postnatal questionnaires there will be follow-up by telephone call at home for non-responders.

### Data management, proposed analyses and reporting of results

#### Data management

The Expected Date of Confinement (EDC) of each participant recruited is entered on an Excel spreadsheet and a register of trial participants is accessed from our hospital patient database (OACIS) on a daily basis on order to identify when a trial participant has delivered. The date of delivery is entered on the spreadsheet which then utilises calculated fields to indicate when 6 week and six month postnatal surveys are due. Data entry onto a computer database, data verification and completion of all data fields will be finalised as early as possible at each time point that the data becomes available. All data will be accounted for and reasons given for why any data is missing.

#### Analyses

All primary and secondary outcomes will be analysed using the "Intention to treat" principle. A comparison of key endpoints will be made for mothers in all three groups. Analyses will be performed with a researcher blinded to group allocations. Initial analyses will examine baseline characteristics of all randomised participants as per the intention to treat principle. If chance differences in baseline data are found between treatment groups, these will be taken into account in subsequent analyses. The population will be characterised by maternal age, highest level of education, maternal expectations of a normal delivery and parity.

#### Statistical tests

Descriptive statistics Mean/sd for parametric data, Median and inter-quartile range for non-parametric data. For Dichotomous outcomes: Chi squared/Relative Risks with 95% confidence intervals. Regression analysis will be utilised to examine the influence of potential confounders on our outcomes of interest. NNT for benefit and harm where appropriate will be calculated.

#### Sub-group analyses

Subgroup analyses of primary outcomes will be performed for women regarding; induced vs spontaneous labour; attendance at all three sessions, whether each session cd was used at least once; size of group < 3 women, hypnotist, women's hypnotisability as measured by the CIS, women's beliefs; on whether they were in experimental group, women's beliefs of the efficacy of hypnosis prior to labour, Women's expectations of requiring an epidural, Women's expectations of having a normal spontaneous birth, Previous experience of non childbirth hypnosis or yoga. hypnotisability as measured by the Creative Imagination Scale. Some women will not attend the sessions to which they are allocated. We have tailored the hypnosis intervention so that most of the skills are learned in the first session and nearly all the skills are delivered by the second session. The third session is for re-enforcement and consolidation. Any women giving birth prior to completing all three sessions will be analysed both on an "intention-to-treat" basis and with total session exposure as a predictor.

#### Interim sequential analyses

Interim sequential analyses as described by Whitehead[64] are planned for all primary and main secondary outcomes when 150 and 300 participants have been recruited. Our independent data monitoring committee (DMC) has clear stopping rules in their terms of reference when reviewing data and will perform analyses as Group A vs B vs C without knowing which group is intervention or control. Stopping rules will include: a significant difference in serious adverse events between groups; clear differences (p < 0.01) between groups shown in primary outcome, and futility in continuing the trial is established. If these criteria are not met the interim sequential analyses will not be revealed to the researchers until the end of the trial. If this is the case the Steering Committee will be informed of the DMC recommendations.

#### Health economic appraisal of hypnosis preparation for childbirth in late pregnancy

Antenatal hypnosis preparation for childbirth may be associated with substantial decreased costs to the health care system and an economic appraisal has been incorporated into the trial from its inception with a view to running a concurrent side study to the main trial. The magnitude of the marginal direct cost of providing the group hypnosis sessions is expected to be low in relation to the total costs of an episode of care. A cost effectiveness analysis is planned that will compare the opportunity cost to society of the additional resources required for the proposed intervention with the consequent gain in health outcome. Direct and indirect costs will be considered including those incurred by the health service, mothers and their immediate families as determined by surveying a subset of trial participants. Resource items will be quantified from the medical record and patient interview. Outcomes such as postpartum hospital readmission within six weeks of discharge; duration of postpartum hospital stay; cost of hospital stay; hospital follow up for long-term morbidity will be calculated.

#### Safety concerns

Women scoring > 12 on Edinburgh Post Natal Depression Score, or where otherwise indicated will be advised by a researcher to see their GP for advice. All participants have consented for treating clinicians to be informed of any clinical concerns and the need for further clinical assessments during the trial.

#### Confidentiality and data security

All patient data will be de-identified during analyses. All trial documentation and participant identifiers will be kept in a locked cabinet and stored for 15 years after publication of the trial results. Trial data on a computer database will be kept password protected.

## Discussion

This trial seeks to determine the efficacy or otherwise of antenatal group hypnosis preparation for childbirth in late pregnancy.

### Evidence for scale validity

#### Measuring pain

Pain is not a directly observable or measurable phenomenon, but rather a subjective experience with sensory and affective elements [65]. In this sense, pain is a psychological phenomenon. Measuring analgesia requirements during labour is a well recognised reliable near objective measure of pain and is commonly used as a *post hoc *measure of pain [7].

#### Psychological testing in pregnancy

The main psychological measures that we plan to use are well-established, validated scales that have been recommended for child-bearing women [36,66]. The Edinburgh Postnatal Depression (EPDS)[61] and the Spielberger anxiety scale[60] will be administered, before the allocated treatments antenatally and in the post-natal period, by researchers blinded to group allocation. The EPDS has been developed to assist primary care health professionals to detect mothers suffering from postnatal depression; a distressing disorder more prolonged than the "blues" (which occur in the first week after delivery) but less severe than puerperal psychosis. Previous studies have shown that postnatal depression affects at least 13% of women and that many depressed mothers remain untreated. These mothers may cope with their baby and with household tasks, but their enjoyment of life is seriously affected and it is possible that there are long-term effects on the family. The EPDS was developed at health centers in Livingston and Edinburgh in the UK. It consists of ten short statements. The mother underlines, which of the four possible responses is closest to how she has been feeling during the past week. Most mothers complete the scale without difficulty in less than 5 minutes. The mother completes the scale herself, unless she has limited English or has difficulty with reading. An EPDS > 12 is considered the level where a need for intervention is clinically indicated [61,67]. The validation study showed that 92.3% mothers who scored above this threshold were likely to be suffering from a depressive illness of varying severity. Other planned measures of "Satisfaction" and "Maternal control" have been utilised previously in pregnancy using Likert scales or visual analogue scores(VAS) [68].

#### Hypnotisability testing

In the laboratory, approximately 14% of people are refractory or uncooperative towards hypnosis, about 36% enter a light hypnotic state, over 25% perform in the moderate range and just under 25% score as highly responsive69 Hypnotisability tests used for research purposes tend to be extensive, can take up to an hour to administer and require a hypnosis induction [70]. Awake suggestibility tests allow for hypnotisability testing of all groups without subjecting women in non-hypnosis groups to a hypnosis induction which may in itself have an effect on our outcomes of interest. The creative imagination scale[62] can be used to test for hypnotisability without a hypnotic induction and has been validated in pregnancy [36]. The administration and scoring of this scale takes approximately 20 minutes to perform. In the clinical setting of stress and pregnancy responsiveness of subjects to hypnosis appears to increase dramatically [71].

#### Blinding

The nature of the intervention makes it difficult to double blind comparisons between hypnosis groups and non intervention controls. However every attempt will be made to conceal treatment allocations from obstetricians, anaesthetists, midwives and those personnel collecting and analysing data. All participants will be informed that they may or may not appreciate which group they are in. Women allocated to usual care will probably realise they are not in an intervention group. Although it may not be possible to blind the administration of our intervention, all data will be collected and analysed by researchers who will be unaware of the participant's group allocation. Any comparisons of participants receiving an intervention in Groups 1 and 2 will be in a true double blind fashion with both participants and outcome assessors blinded to allocation. Our primary outcomes and key secondary outcomes are designed to be as objective as they can be for a study of this type. An assessment of blinding will be determined by asking participants if they thought they were in a control or intervention group in the final post-partum questionnaire.

There are four main reasons that provide the rationale for a clinical hypnosis study in late pregnancy. Firstly, pain and the fear of pain associated with childbirth is nearly universal and currently utilised pharmacological methods of pain relief have limitations and well recognised complications associated with their use. Confirming the association of reducing pharmacological analgesia requirements in labour will reduce the incidence of their complications and potentially improve the childbirth experience. Hypnosis has been shown to effectively provide anxiolysis and analgesia in a range of clinical[28,72,73] and laboratory settings [30,39,69,74]. Secondly, we have found that our hypnosis intervention is easy to administer and appears to be a relatively simple and inexpensive means of preparing women for labour and childbirth. Hypnosis has a long history of use in childbirth which is claimed to be one of the most useful settings in which to utilise hypnosis [23,75,76]. Systematic reviews of the evidence to date[10,22,43] and our use of hypnosis as antenatal preparation for labour[47,77] suggest benefits in: reducing analgesia requirements; reducing the incidence of oxytocin administration; and increasing the incidence of spontaneous vaginal birth. However the existing evidence from small or poorly designed trials is still inadequate to confirm these effects. Renewed interest in this topic and the call for more research is as relevant today as it was 30 years ago [78]. Third, this is the first randomised trial investigating the effects of antenatal hypnosis preparation for childbirth in late pregnancy in both nulliparous and multiparous women; with a structured defined intervention that is easily reproducible and has excellent external validity; with clearly described adequate allocation concealment; and with the incidence of postnatal depression as a key endpoint. Fourth, peri-operatively the use of hypnosis has been shown to increase the incidence of beneficial outcomes and lower costs. This is likely to be translated into the childbirth setting.

## Competing interests

The author(s) declare that they have no competing interests.

## Authors' contributions

AMC, MIA, JSR, CAC have contributed to overall concept and design. AMC, MIA, GW and CW designed and co-ordinated the final content of the hypnotherapeutic interventions and hypnotisability testing. AMC, DT, MIA designed the implementation of psychological testing. AMC and PB have organised acquisition of data and analysis of data. AMC, MIA, JSR, CAC, PB, were involved in drafting the manuscript. Each author has participated sufficiently in the work to take public responsibility for appropriate portions of the content. AMC has been responsible for acquiring funding and has supervised the research. All authors have read and approved the final version of the manuscript.

## Pre-publication history

The pre-publication history for this paper can be accessed here:



## Supplementary Material

Additional File 1 **Detailed hypotheses** Click here for file

Additional File 2 **Rate differences calculated in treatment and control groups for Key endpoints with a sample size of 135/group. **Nullip. = Nulliparous, Multip. = Multiparous Click here for file
