# Supplementary material for: Hypnosis Antenatal Training for Childbirth (HATCh): a randomised controlled trial [NCT00282204]
Source: BMC Pregnancy Childbirth. 2006 Mar 5;6:5. doi: 10.1186/1471-2393-6-5 (PMC1450315; doi:10.1186/1471-2393-6-5)
Supplement: Additional File 1 — Detailed hypotheses [file 1471-2393-6-5-S1.doc]

| Hypotheses |  |
| --- | --- |
| Primary hypothesis | Antenatal hypnosis when compared with no intervention reduces the use of pharmacological analgesia during labour, in mothers planning a normal vaginal birth, as measured by documentation of analgesia techniques used in the birth register or maternal medical record |
| Null hypothesis | Antenatal hypnosis has no effect on maternal pharmacological analgesia requirements when compared with no intervention |
| Secondary hypotheses | Antenatal hypnosis when compared with no intervention:-   - decreases the maternal perception of the overall pain experienced during labour and childbirth as measured by a 0-10 numerical rating scale in the postnatal period within one week and at 6 weeks and 6 months; - increases the incidence of mothers reporting that they received adequate pain relief during childbirth as measured by postnatal maternal questionnaire; increases the incidence of spontaneous vaginal birth as measured by documentation in the birth register or medical record; - decreases the use of oxytocics as documented in the birth register or medical record; - decreases the duration of labour as measured by documentation in the birth register or medical record; - increases maternal satisfaction with the birth experience as measured by maternal questionnaires that include a 0-10 numerical rating scale in the postnatal period within the first week and at 6 weeks and 6 months; - increases a perceived sense of maternal control during the labour and during the birth as measured by maternal questionnaires that include a 0-10 numerical rating scale within one week of the birth; increases the mother’s subjective ability to cope with labour / childbirth as measured by maternal questionnaires that include a 0-10 numerical rating scale in the postnatal period within one week of the birth; - increases the incidence of a positive birth experience as measured by maternal questionnaires that include a 3 point verbal descriptor scale within one week of the birth; - decreases the incidence of admission of the baby to the neonatal unit as measured by the medical and nursing notes; - decreases baby length of stay in hospital as measured by the medical and nursing notes; - decreases maternal length of stay in hospital as measured by the maternal record documentation; - increases the incidence of breast feeding post partum as measured by the mothers response to the postnatal questionnaires at 6 weeks and 6 months; decreases the incidence of postnatal depression as measured by the mothers response to the Edinburgh Postnatal Depression Scale (EPDS) and specific questions about being treated for postnatal depression in the postnatal questionnaires at 6 weeks and 6 months; - decreases post partum anxiety as measured by the mothers response to the Spielberger Stait/Trait Anxiety Scale in postnatal questionnaires at 6 weeks and 6 months; decreases average hospital costs compared with no intervention as measured by estimating average hospital costs of each group based on length of hospital stay/cost of treatments |
| Other Secondary outcomes: | Antenatal hypnosis guided by a hypnotherapist has a greater treatment effect on primary and secondary outcomes, when compared with an audio cd on hypnosis administered by a nurse with no hypnotherapy training, as measured by the maternal / birth record documentation and the mothers’ responses to our postnatal questionnaires.  Hypnotisability as measured by the Creative Imagination Scale is predictive of analgesia requirements in childbirth, and other maternal / neonatal / birth outcomes.  Antenatal expectations of the need for epidural analgesia and predicting a spontaneous vaginal birth, as measured by maternal response to baseline antenatal questionnaire, is predictive of analgesia requirements in childbirth, and other maternal / neonatal / birth outcomes.  Cost - Benefit analyses favours hypnosis compared with no intervention as measured by treatment costs and their impact on hospital costs and the value to patients of health outcome impacts |
