# Supplementary material for: Hypnosis Antenatal Training for Childbirth (HATCh): a randomised controlled trial [NCT00282204]
Source: BMC Pregnancy Childbirth. 2006 Mar 5;6:5. doi: 10.1186/1471-2393-6-5 (PMC1450315; doi:10.1186/1471-2393-6-5)
Supplement: Additional File 2 — Rate differences calculated in treatment and control groups for Key endpoints with a sample size of 135/group. Nullip. = Nulliparous, Multip. = Multiparous [file 1471-2393-6-5-S2.doc]

| Key endpoints | Rate for usual care % | Expected % change detectable by sample size | Expected Rate level for hypnosis women given this % change |
| --- | --- | --- | --- |
| Pharmacological analgesia* | 80 | 20 | 64 |
| Epidural rate** | Total 39  Nullip.=62  Multip. = 33 | Total=41  Nullip. = 27  Multip. = 45 | Total=23  Nullip.=45  Multip. = 18 |
| Use of oxytocics** | Total = 31  Nullip. = 36  Multip. = 18 | Total = 48  Nullip. = 45  Multip. = 65 | Total = 16  Nullip. = 20  Multip. = 6 |
| Spontaneous vaginal birth** | Total = 57  Nullip. = 47.5  Multips = 77 | Total 26  Nullip.35  Multip.18 | Total 73  Nullip.65  Multip.91 |
| Postnatal depression | 16 | 69 | 5 |
| EPDS | Unknown |  |  |
| Spielberger | Unknown |  |  |

* Data collected from the Birth register at our institution for the month of May 2004

* * Clinical Information Service 2004 data for our institution
